# Isolation of Human Intestinal Bacteria Capable of Producing the Bioactive Metabolite Isourolithin A from Ellagic Acid

**DOI:** 10.3389/fmicb.2017.01521

**Published:** 2017-08-07

**Authors:** María V. Selma, David Beltrán, María C. Luna, María Romo-Vaquero, Rocío García-Villalba, Alex Mira, Juan C. Espín, Francisco A. Tomás-Barberán

**Affiliations:** ^1^Laboratory of Food and Health, Research Group on Quality, Safety and Bioactivity of Plant Foods, Department of Food Science and Technology, Centre for Applied Soil Science and Biology of the Segura – Spanish National Research Council Murcia, Spain; ^2^Department of Health and Genomics, Center for Advanced Research in Public Health, FISABIO Foundation Valencia, Spain

**Keywords:** urolithin, ellagitannin, bioconversion, metabotype, novel probiotic, gut bacteria, polyphenols

## Abstract

Urolithins are intestinal microbial metabolites produced from ellagitannin- and ellagic acid-containing foods such as walnuts, strawberries, and pomegranates. These metabolites, better absorbed than their precursors, can contribute significantly to the beneficial properties attributed to the polyphenols ellagitannins and ellagic acid (EA). However, both the ability of producing the final metabolites in this catabolism (urolithins A, B and isourolithin A) and the health benefits associated with ellagitannin consumption differ considerably among individuals depending on their gut microbiota composition. Three human urolithin metabotypes have been previously described, i.e., metabotype 0 (urolithin non-producers), metabotype A (production of urolithin A as unique final urolithin) and metabotype B (urolithin B and/or isourolithin A are produced besides urolithin A). Although production of some intermediary urolithins has been recently attributed to intestinal species from *Eggerthellaceae* family named *Gordonibacter urolithinfaciens* and *Gordonibacter pamelaeae*, the identification of the microorganisms responsible for the complete transformation of EA into the final urolithins, especially those related to metabotype B, are still unknown. In the present research we illustrate the isolation of urolithin-producing strains from human feces of a healthy adult and their ability to transform EA into different urolithin metabolites, including isourolithin A. The isolates belong to a new genus from *Eggerthellaceae* family. EA transformation and urolithin production arisen during the stationary phase of the growth of the bacteria under anaerobic conditions. The HPLC-DAD-MS analyses demonstrated the sequential appearance of 3,8,9,10-tetrahydroxy-urolithin (urolithin M6), 3,8,9-trihydroxy-urolithin (urolithin C) and 3,9-dihydroxy-urolithin (isourolithin A) while 3,8-dihydroxy-urolithin (urolithin A) and 3-hydroxy-urolithin (urolithin B) were not detected. For the first time isourolithin A production capacity of pure strains has been described. The biological activity attributed to urolithins A and B and isourolithin A (anti-inflammatory, anti-carcinogenic, cardioprotective, and neuroprotective properties) explains the relevance of identifying these urolithin-producing bacteria as potential novel probiotics with applications in the development of functional foods and nutraceuticals. Their human administration could improve the health benefits upon ellagitannin consumption, especially in metabotype 0 individuals. However, further research is necessary to probe well-established beneficial effects on the host and safety requirements before being considered among the next-generation probiotics.

## Introduction

Ellagitannins and EA produce some health benefits through the consumption of walnuts, strawberries, and pomegranates among other fruits (Tomás-Barberán et al., 2016a). Their bioavailability is low but it is now well-established that they are transformed by the intestinal bacteria to urolithins that are better absorbed (Cerdá et al., 2004). Identification of the urolithins released from dietary ellagitannins by intestinal microbiota is a present tendency in phenolic investigation because of the health implication of these microbial metabolites as potential anti-inflammatory, antioxidant, cardioprotective, neuroprotective, and cancer preventive compounds (Larrosa et al., 2010; Espín et al., 2013). However, both the health benefits associated with ellagitannin consumption and the ability of producing urolithins in this catabolism differ considerably among individuals. Population has been categorized into three ellagitannin-metabolizing phenotypes, i.e., ‘urolithin metabotypes,’ depending on the quantitative percentage and type of the urolithins formed. Thus, metabotype A is distinguished by the production of urolithin A, metabotype B individuals produce isourolithin A and urolithin B besides urolithin A, and those with metabotype 0 do not produce the final metabolites urolithin A, isourolithin A, or urolithin B. This interindividual variability has been related with dissimilarities in the intestinal microbiota (Tomás-Barberán et al., 2014; Romo-Vaquero et al., 2015; Selma et al., 2015). Therefore, the identification of the microbial species able to transform the ellagitannins, EA and other polyphenols is also an important goal because of the possible development of functional foods with health benefits on low producers of urolithins (Tomás-Barberán et al., 2016b; Espín et al., 2017). The bacterial species responsible for urolithin production are scarcely known. Only two urolithin-producing species *Gordonibacter pamelaeae* (DSM 19378^T^) and *Gordonibacter urolithinfaciens* (DSM 27213^T^) have been identified as producers of intermediary urolithins (Selma et al., 2014a,b). Therefore, other still unknown bacteria are necessary for the complete transformation of EA into the final metabolites (urolithin A, isourolithin A, or urolithin B). In the present research we illustrate for the first time the isolation of an urolithin-producing bacteria from human feces from a healthy adult, its phylogenetic analysis and its ability to transform EA into different urolithin metabolites including the final metabolite isourolithin-A.

## Materials and Methods

### Isolation of Urolithin-Producing Bacteria

A healthy male donor (aged 41), who previously demonstrated to produce urolithins *in vivo,* provided the stool samples. The study was conformed to ethical guidelines outlined in the Declaration of Helsinki and its amendments. The protocol (included in the project AGL2015-64124-R) was approved by the Spanish National Research Council’s Bioethics Committee (Spain). Donor gave written informed consent in accordance with the Declaration of Helsinki. Urolithins were identified in feces and urine after walnut consumption as explained elsewhere (Selma et al., 2016). The feces were prepared for isolation of microorganisms following the isolation protocol previously described with some modifications (Selma et al., 2014a,b). Briefly, after 1/10 (w/v) fecal dilution in nutrient broth (Oxoid, Basingstoke, Hampshire, United Kingdom) supplemented with 0.05% L-cysteine hydrochloride (PanReac Química, Barcelona, Spain), the filtrated was homogenized and further diluted in ABB (Oxoid). In order to first evaluate the metabolic activity, 15 μM urolithin C (Dalton Pharma Services, Toronto, ON, Canada) was dissolved in propylene glycol (PanReac Quiìmica SLU, Barcelona, Spain) and added to the broth. After anaerobic incubation, a portion of the culture, having metabolic activity, was seeded on ABB agar. Approximately 200 colonies were collected, inoculated into 5 ml of ABB containing EA (Sigma–Aldrich, St. Louis, MO, United States) at 15 μM and after incubation; their capacity to convert EA into urolithins was assayed. Urolithin-producing colonies were sub-cultured until urolithin-producing strains were isolated. The isolation procedure and plate incubation was achieved under anoxic environment with an atmosphere consisting of N_2_/H_2_/CO_2_ (85/5/10) in an anaerobic chamber (Concept 400, Baker Ruskin Technologies, Ltd, Bridgend, South Wales, United Kingdom) at 37°C. Samples (5 ml) were prepared for HPLC-DAD-MS analyses of urolithins as explained below.

### Phylogenetic Classification of the Urolithin-Producing Bacteria

The almost-complete 16S rRNA gene sequence of the isolated strains was obtained by PCR amplification on a AG 22331 thermocycler (Eppendorf), followed by direct sequencing using primers 616V (forward) and 699R (reverse), as described before (Arahal et al., 2008) to target about 1000 nt close to the 5′ end and primers P609D and P1525R to target positions 785–802 and 1525–1541, respectively, as previously described (Lucena et al., 2010). The resulting amplicons were analyzed by Sanger sequencing. Sequencing data were assessed using Lasergene (DNASTAR), were manually corrected and compared with public sequences in the EMBL database using the BLAST program (National Center for Biotechnology Information^1^). The phylogenetic analysis was performed with MEGA7: Molecular Evolutionary Genetics Analysis version 7.0 for bigger datasets (Kumar et al., 2016). Neighbor-joining treeing method was used and distance matrix was calculated by the Jukes and Cantor method (Jukes and Cantor, 1969). The data subsets were performed using the appropriate MEGA 7 tools.

### Growth Kinetics and Time-Course Transformation of Ellagic Acid and Urolithin C

An isolated strain with the ability to produce isourolithin A, preserved frozen, was incubated on ABB agar plate for 6 days. A single colony was cultivated in 5 ml ABB tube. Two milliliters of diluted inoculum were transferred to ABB (200 ml) obtaining an initial load of 10^4^ cfu ml^-1^. Separately, EA or urolithin C, dissolved in propylene glycol, were added to the 200 ml cultures to obtain a final concentration of 15 μM. During incubation in anoxic environment at 37°C, aliquots (5 ml) were taken for HPLC analyses as described below. Plate counts in ABB agar were carried out. Growth curves were made in triplicate and the experiment was repeated three times.

### HPLC-DAD-MS Analyses

Aliquots collected during the incubation of isolated strains, were extracted and analyzed by HPLC-DAD-ESI-Q (MS) as previously described (García-Villalba et al., 2016). Briefly, fermented medium (5 ml) was extracted with ethyl acetate (5 ml) (Labscan, Dublin, Ireland) acidified with 1.5% formic acid (Panreac), vortexed for 2 min and centrifuged at 3500 *g* for 10 min. The organic phase was separated and evaporated and the dry samples were then re-dissolved in methanol (250 μl) (Romil, Barcelona, Spain). An HPLC system (1200 Series, Agilent Technologies, Madrid, Spain) equipped with a photodiode-array detector (DAD) and a single quadrupole mass spectrometer detector in series (6120 Quadrupole, Agilent Technologies, Madrid, Spain) was used as previously described (García-Villalba et al., 2016). Calibration curves were obtained for EA, urolithin C as well as urolithin M6 and isourolithin A (Villapharma SL, Murcia, Spain) with good linearity (*R*^2^ > 0.998). Isourolithin A and urolithin C were quantified at 305 nm, while urolithin M5, urolithin M6, and EA were quantified at 360 nm, all with their own standards.

### Data Modeling of Growth Curves

Bacterial growth curves and main growth parameters (lag time, maximum specific growth rate, and estimated correlation coefficient) were fitted with the function of Baranyi et al. (1993).

## Results

### Identification of Urolithin Producing Bacteria

**FIGURE 1:**
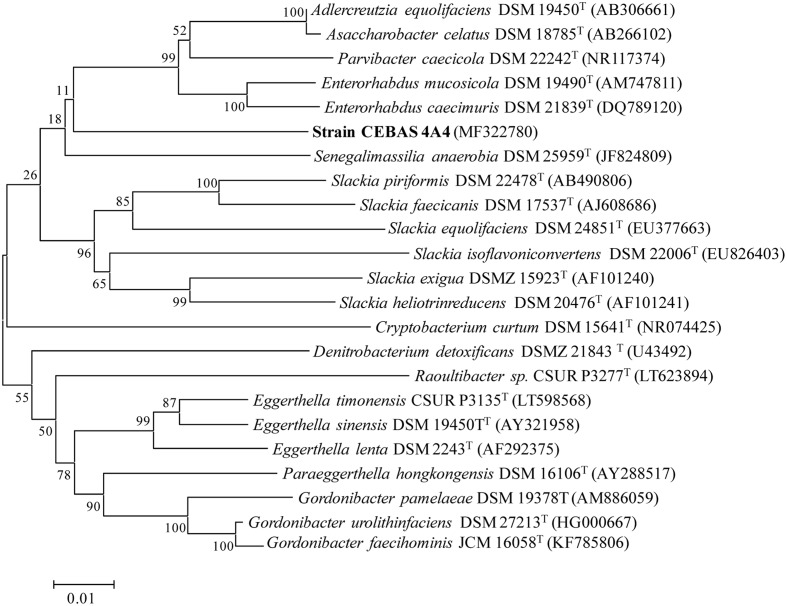
Phylogenetic tree showing the relationship between the strain CEBAS 4A4 and other representatives of the family *Eggerthellaceae*. The tree was constructed by using the neighbor-joining method based on 16S rRNA gene sequences. Distance matrix was calculated by the Jukes and Cantor method. GenBank accession numbers are presented in parentheses. Bar, 1% nucleotide sequence difference. Numbers at nodes (≥70%) indicate support for internal branches within the tree obtained by bootstrap analysis (percentages of 500 re-samplings).

Enrichment cultures resulted in the isolation of four pure bacterial cultures (strains CEBAS 4A1, 4A2, 4A3, 4A4) which showed the capacity to convert EA into isourolithin A as final metabolite under anaerobic conditions. The 16S rRNA gene sequence of the isolates showed 100% similarity and one of the isolates (strain CEBAS 4A4) has been deposited in the NCBI nucleotide sequence database under accession number MF322780. The sequence of the 16S rRNA gene and phylogenetic characteristics from more closely related species showed that strain CEBAS 4A4 belonged to the family *Eggerthellaceae* (**Figure 1**). Strain CEBAS 4A4 has been deposited in two public culture collections (= DSM 104140^T^ = CCUG 70284^T^).

### Analysis of Urolithins Produced by the Strain CEBAS 4A4

**FIGURE 2:**
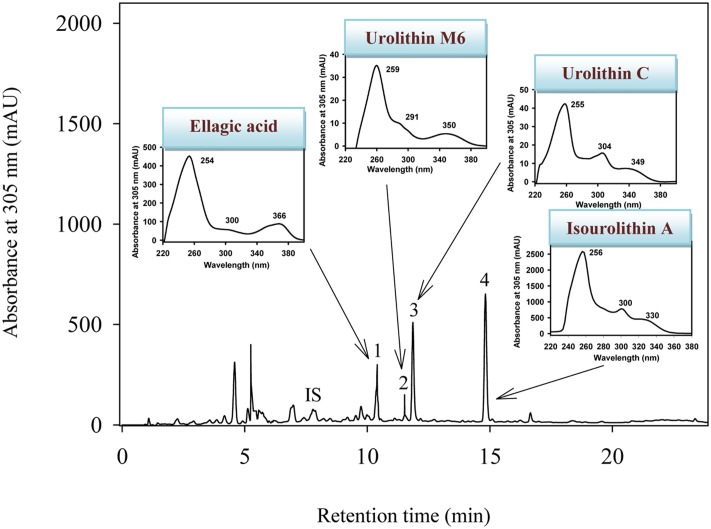
HPLC-DAD-MS elution profile of *in vitro* metabolism of EA by the strain CEBAS 4A4 under anaerobic conditions. The insets show the UV spectra of EA and its metabolites. IS (internal standard; 6,7-dihydroxycoumarin), (1) EA, (2) 3,8,9,10-tetrahydroxy-urolithin (urolithin M6), (3) 3,8,9-trihydroxy-urolithin (urolithin C), (4) 3,9-dihydroxy-urolithin (isourolithin A).

The HPLC-DAD-MS analyses showed that urolithin M6, urolithin C, and isourolithin A were produced from EA by all the isolated strains (CEBAS 4A1, 4A2, 4A3, 4A4) (**Figure 2**). Identification of metabolites was carried out by direct comparison (MS and UV spectra) with pure standards and confirmed by their molecular mass and spectra (García-Villalba et al., 2016).

### Growth Kinetics and Time-Course Catabolism of EA and Urolithin C by the Strain CEBAS 4A4

**FIGURE 3:**
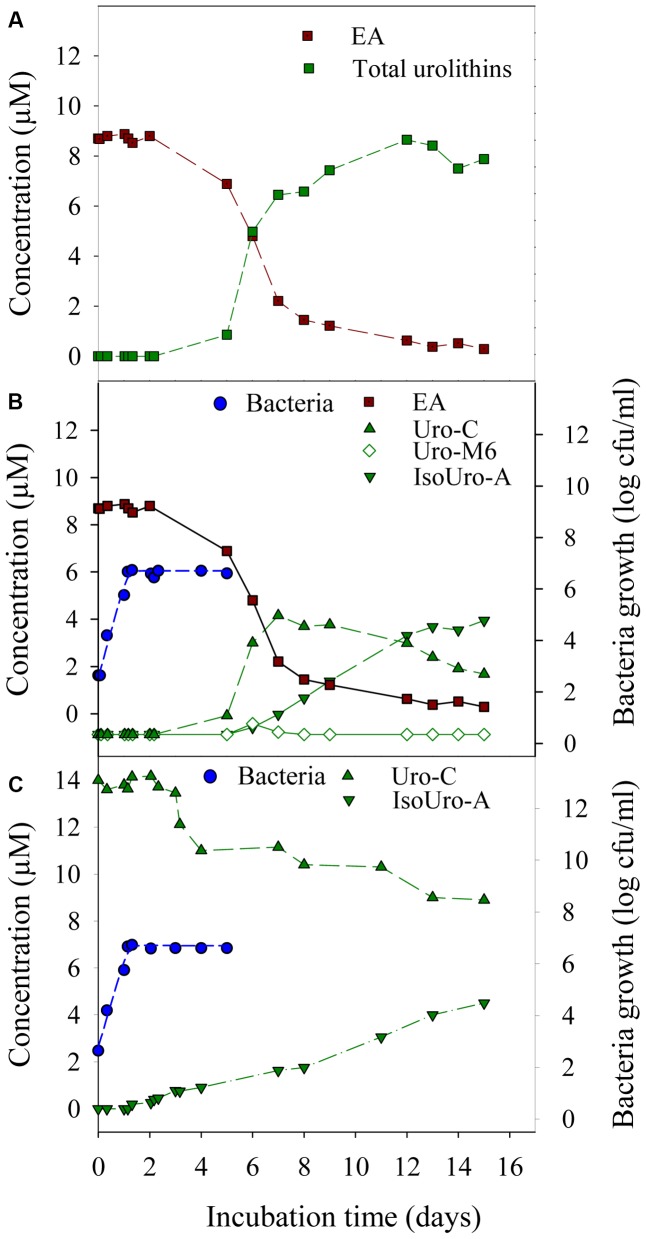
Bacterial growth and time course production of urolithins by the strain CEBAS 4A4. Metabolism of EA to total urolithins **(A)** and to isourolithin A (IsoUro-A) via urolithin M6 (Uro-M6) and urolithin C (Uro-C) **(B)**. Metabolism of urolithin C (Uro-C) to isourolithin-A (IsoUro-A) in absence of EA **(C)**.

The lag phase of growth for strain CEBAS 4A4 was 1.5 ± 0.6 h while the growth rate was 0.15 ± 0.01 h^-1^ with and without EA or with and without urolithin C at 15 μM. EA and urolithin C catabolism and isourolithin A production took place during the stationary phase of the growth (**Figure 3**). EA disappeared simultaneously that urolithins appeared (**Figure 3A**). Urolithin M5 was not detected while urolithin M6 was only observed in the sample obtained at day 6, being the first metabolite detected. Urolithin C arrived to a maximum at day 7, and then diminished progressively whereas isourolithin A was formed. The full conversion of EA into urolithin C (35%) and isourolithin A (65%) was reached at day 13 while urolithin A was not detected (**Figures 3A,B**). A longer incubation up to 15 days did not produce further hydroxyl removals from isourolithin A. Therefore, 3-hydroxy-urolithin (urolithin B) was not detected. In contrast to *in vitro* catabolism of EA, the complete transformation of urolithin C into isourolithin A was not obtained during incubation with the strain CEBAS 4A4 (**Figure 3C**). After 15 days, the maximum conversion of EA into isourolithin A was 33%.

## Discussion

The percentage of metabotype B in adult healthy population ranges from 20 to 30%, and it is mainly characterized by isourolithin A and/or urolithin B production as final urolithins (Tomás-Barberán et al., 2014). However, the gut bacteria able to produce these metabolites have not been described so far. Four gut bacteria strains (CEBAS 4A1, 4A2, 4A3, 4A4) isolated from a healthy donor and able to produce urolithins M6, C and isourolithin A, are described in the present study. Strains were found at high concentrations (≥10^7^ cfu g^-1^ feces). Comparison of the 16S rRNA gene sequences of the strains showed that the four isolates belong to the same species and that they are phylogenetically members of the family *Eggerthellaceae.* Recently, the class Coriobacteriia containing the family *Coriobacteriaceae* has been divided into the Eggerthellales ord. nov. (including the family *Eggerthellaceae* fam. nov.) and the emended order Coriobacteriales (including the emended family *Coriobacteriaceae* and *Atopobiaceae* fam. nov.) (Gupta et al., 2013). Based on 16S rRNA gene sequence, the closest relatives of isolated strain CEBAS 4A4 from the *Eggethellaceae* family includes *Enterorhabdus musicola* DSM 19490^T^ and *Enterorhabdus caecimuris* DSM 21839T (93.0% identity), *Adlercreutzia equolifaciens* DSM 19450^T^ (93.0% identity), *Asaccharobacter celatus* DSM 18785^T^ (92.0% identity), and *Parvibacter caecicola* DSM 22242^T^ (91.0% identity). Even if it is not possible to differentiate species by reason of 16S rRNA sequence differences only, at the present is in general established that bacteria showing > 5% 16S rRNA gene sequence difference are of different genus (Stackebrandt and Goebel, 1994; Fournier et al., 2015). Results of the phylogenetic analysis suggest that isolated strains belong to a novel genus and further analyses describing these bacteria as novel genus and species are being carried out for publication.

Previous studies have identified bacterial species able to transform different polyphenols such as flavan-3-ols and isoflavones to simpler bioactive molecules (Braune and Blaut, 2016). *Eggerthella lenta* and *A. equolifaciens* are able to dehydroxylate flavan-3-ols and their C-ring cleavage products at the B-ring. Transformation of isoflavones to equol have been also associated to *E. musicola, A. equolifaciens, A. celatus, Slackia isoflavoniconvertens, Slackia equolifaciens* (Matthies et al., 2008; Thawornkuno et al., 2009; Braune and Blaut, 2016). There is much less information in relation to the bacteria responsible for the transformation of ellagitannins. Only urolithin C-producing bacteria have been described so far (*G. urolithinfaciens* and *G. pamelaeae*) (Selma et al., 2014a,b). Similarities of 16S rRNA gene sequence of strain CEBAS 4A4 are 91% (76% cover) with *G. pamelaeae* (DSM 19378^T^) and 90% (73% cover) with *G. urolithinfaciens* (DSM 27213^T^). All these bacteria are members of the *Eggerthellaceae* family although they were previously considered from *Coriobacteriaceae* family. Therefore, as a result of the recent division of the class Coriobacteriia, bacterial species described as polyphenol transformers have been regrouped within the Eggerthellales ord. nov. while phylogenetic neighbors, not associated with the transformation of polyphenols (*Collinsella, Atopobium,* and *Olsenella* genera), are grouped in the emended order Coriobacteriales. Further studies should be performed to elucidate the potential health benefits of bacteria from *Eggerthellaceae* family due to their capacity to produce bioactive molecules from dietary polyphenols.

**FIGURE 4:**
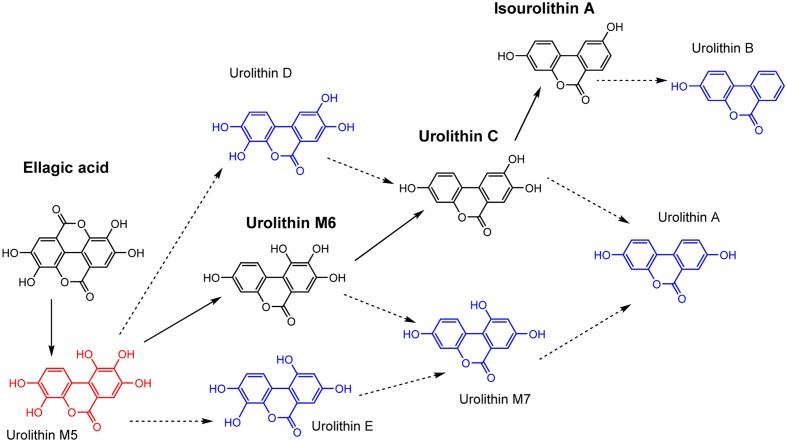
Catabolic pathway for EA by intestinal bacteria. Proposed metabolic pathway of EA by strain CEBAS 4A4 (black color). Other urolithins which are not produced by strain CEBAS 4A4 (blue color).

In the current research, the chronological production of urolithins M6, C and isourolithin A by isolated strains (CEBAS 4A1, 4A2, 4A3, 4A4) has been shown (**Figure 4**). Although isolated strains share with *G. urolithinfaciens* and *G. pamelaeae* (Selma et al., 2014a,b) the ability to produce urolithin C, strains CEBAS 4A1, 4A2, 4A3, 4A4 are able to produce a further dehydroxylation to yield the final metabolite isourolithin A. Previous studies identified urolithins A, B, C and isourolithin A in human urine, plasma and target tissues such as the colon and prostate (González-Sarrías et al., 2010, 2016; Nuñez-Sánchez et al., 2014). Production of urolithins M5, M6 and M7, E, C, A, B and isourolithin A by human fecal microbiota has also been described *in batch culture* (García-Villalba et al., 2013) and in a intestinal simulator (TWIN-SHIME^®^) (García-Villalba et al., 2017). However, it is in the present study where one bacterial species is identified as producer of isourolithin A. Accordingly, other intestinal species are needed for producing urolithins A and B. Additional research should be performed to find out if the deficiency of the isolated bacteria described in the present study is the restrictive factor in the formation of isourolithin A *in vivo*. In the case of the bacteria involved in the formation of isourolithin A, and according to the catabolic pathway of EA to yield urolithins (**Figure 4**), it is remarkable the specific *o*-dehydroxylase activity displayed by this microorganism. Whereas *G. urolithinfaciens* has been reported to *o*-dehydroxylate urolithin M6 to yield urolithin C (Selma et al., 2014b) however, it was not able to catalyze further dehydroxylations. In this case, the strain CEBAS 4A4 selectively and sequentially can *o*-dehydroxylate both urolithins M6 and C to yield isourolithin A but it was not able to catalyze the dehydroxylation of the hydroxyl group at the *para* position (**Figure 4**). This is somehow paradoxical as this hydroxyl group, located at the 9-position, has been reported to be more reactive than the hydroxyl group at the 8-position (González-Sarrías et al., 2017). Indeed, the ionization of the phenolic hydroxyl at 9-position is preferential against the hydroxyl at 8-position in urolithin A, which indicates that the hydroxyl at 9-position is more acidic and thus can perhaps be a better substrate for a number of enzymes (González-Sarrías et al., 2017).

Complete transformation of EA (8 μM) into urolithins by isolated strain CEBAS 4A4 (13 days) or *G. urolithinfaciens* was produced after 7 days, being urolithin C the major metabolite. However, 5 more days were needed to achieve the highest concentration of isourolithin A in the case of isolated strain CEBAS 4A4 while *G. urolithinfaciens* did not produce further dehydroxylations from urolithin C. Urolithins produced by strain CEBAS 4A4 or by *Gordonibacter* species are secondary metabolites without role in the bacterial growth because the stationary phase of growth is achieved before urolithin production. In opposition to synthetic chemistry, metabolite production by microorganisms is often more convenient (Craney et al., 2013). Bacterial secondary metabolites, such as antibiotics, antitumorals, cholesterol-lowering agents, immunomodulating agents are being progressively used more in diseases treated only by synthetic medicines in the past. In addition to the classical probiotics (*Lactobacillus* and *Bifidobacterium*), other beneficial microbiota such as the butyrate-producing *Faecalibacterium prausnitzii* (Eppinga et al., 2016) and the mucin-degrading *Akkermansia muciniphila* (Derrien et al., 2016) have recently been described. The urolithin-producing bacteria described herein, in addition to other dietary polyphenol-transforming bacteria, could also have potential as novel probiotics as well as in the industrial manufacture of bioactive metabolites to develop new ingredients, beverages, nutraceuticals, pharmaceuticals, and/or functional foods. However, further studies should be carried out to demonstrate these emerging points.

## Conclusion

We report here the isolation of human gut bacterial strains that belong to a new genus from *Eggerthellaceae* family. This is the first description of bacterial strains capable of converting *in vitro* EA into the final metabolite isourolithin-A. The human health benefits associated with urolithins explain the relevance of identifying the responsible gut bacteria potentially useful for the development of novel probiotics, functional foods, and food complements. This is especially relevant in those individuals with metabotype 0, who are not able to produce bioactive urolithins. However, further research is necessary to probe well-established health effects on the host as well as safety requirements before being considered among the next-generation probiotics.

## Author Contributions

DB, ML, and RG-V performed most of the laboratory work including the isolation of the isourolithin producing bacteria. MR-V and AM performed the phylogenetic analysis of the isolated bacteria. MS established anaerobic experiment conditions and wrote the manuscript. FT-B and JE established HPLC experimental conditions. MS, FT-B, and JE were the project leaders who formed the idea and supervised the work.

## Conflict of Interest Statement

The authors declare that the research was conducted in the absence of any commercial or financial relationships that could be construed as a potential conflict of interest.

## Footnotes

1 http://ncbi.nlm.nih.gov/

## Abbreviations

ABB: anaerobic basal broth
EA: ellagic acid.

## References

[B1] Arahal D. R. Sánchez E. Macián M. C. Garay E. (2008). Value of *recN* sequences for species identification and as a phylogenetic marker within the family “Leuconostocaceae”. *Int. Microbiol.* 11 33–39. 10.2436/20.1501.01.42 18683630

[B2] Baranyi J. Roberts T. A. McClure P. (1993). A non-autonomous different equation to model bacterial growth. *Food Microbiol.* 10 43–59. 10.1006/fmic.1993.1005

[B3] Braune A. Blaut M. (2016). Bacterial species involved in the conversion of dietary flavonoids in the human gut. *Gut Microbes* 7 1–19. 10.1080/19490976.2016.1158395 26963713 PMC4939924

[B4] Cerdá B. Espín J. C. Parra S. Martínez P. Tomás-Barberán F. A. (2004). The potent in vitro antioxidant ellagitannins from pomegranate juice are metabolised into bioavailable but poor antioxidant hydroxy-6H-dibenzopyran-6-one derivatives by the colonic microflora of healthy humans. *Eur. J. Nutr.* 43 205–220. 10.1007/s00394-004-0461-7 15309440

[B5] Craney A. Ahmed S. Nodwell J. (2013). Towards a new science of secondary metabolism. *J. Antibiot.* 66 387–400. 10.1038/ja.2013.25 23612726

[B6] Derrien M. Belzer C. de Vos W. M. (2016). *Akkermansia muciniphila* and its role in regulating host functions. *Microb. Pathog.* 106 171–181. 10.1016/j.micpath.2016.02.005 26875998

[B7] Eppinga H. Weiland C. J. S. Thio H. B. van der Woude C. J. Nijsten T. E. Peppelenbosch M. P. (2016). Similar depletion of protective *Faecalibacterium prausnitzii* in psoriasis and inflammatory bowel disease, but not in hidradenitis suppurativa. *J. Crohns. Colitis.* 10 1067–1075. 10.1093/ecco-jcc/jjw070 26971052

[B8] Espín J. C. González-Sarrías A. Tomás-Barberán F. A. (2017). The gut microbiota: a key factor in the therapeutic effects of (poly)phenols. *Biochem. Pharmacol.* 17 30252–30256. 10.1016/j.bcp.2017.04.033 28483461

[B9] Espín J. C. Larrosa M. García-Conesa M. T. Tomás-Barberán F. (2013). Biological significance of urolithins, the gut microbial ellagic Acid-derived metabolites: the evidence so far. *Evid. Based Complement. Alternat. Med.* 2013:270418 10.1155/2013/270418 PMC3679724 23781257

[B10] Fournier P.-E. Rossi-Tamisier M. Benamar S. Raoult D. (2015). Cautionary tale of using 16S rRNA gene sequence similarity values in identification of human-associated bacterial species. *Int. J. Syst. Evol. Microbiol.* 65 1929–1934. 10.1099/ijs.0.000161 25736410

[B11] García-Villalba R. Beltrán D. Espín J. C. Selma M. V. Tomás-Barberán F. A. (2013). Time course production of urolithins from ellagic acid by human gut microbiota. *J. Agric. Food Chem.* 61 8797–8806. 10.1021/jf402498b 23984796

[B12] García-Villalba R. Espín J. C. Tomás-Barberán F. A. (2016). Chromatographic and spectroscopic characterization of urolithins for their determination in biological samples after the intake of foods containing ellagitannins and ellagic acid. *J. Chromatogr. A* 1428 162–175. 10.1016/j.chroma.2015.08.044 26341594

[B13] García-Villalba R. Vissenaekens H. Pitart J. Romo-Vaquero M. Espín J. C. Grootaert C. (2017). The gastrointestinal simulation model TWIN-SHIME shows differences between human urolithin-metabotypes in gut microbiota composition, pomegranate polyphenol metabolism, and transport along the intestinal tract. *J. Agric. Food Chem.* 65 5480–5493. 10.1021/acs.jafc.7b02049 28616977

[B14] González-Sarrías A. García-Villalba R. Romo-Vaquero M. Alasalvar C. Orem A. Zafrilla P. (2016). Clustering according to urolithin metabotype explains the interindividual variability in the improvement of cardiovascular risk biomarkers in overweight-obese individuals consuming pomegranate: A randomised clinical trial. *Mol. Nutr. Food Res.* 61:1600830 10.1002/mnfr.201600830 27879044

[B15] González-Sarrías A. Giménez-Bastida J. A. García-Conesa M. T. Gómez-Sánchez M. B. García-Talavera N. V. Gil-Izquierdo A. (2010). Occurrence of urolithins, gut microbiota ellagic acid metabolites and proliferation markers expression response in the human prostate gland upon consumption of walnuts and pomegranate juice. *Mol. Nutr. Food Res.* 54 311–322. 10.1002/mnfr.200900152 19885850

[B16] González-Sarrías A. Nuñez-Sánchez M. A. García-Villalba R. Tomás-Barberán F. A. Espín J. C. (2017). Antiproliferative activity of the ellagic acid-derived gut microbiota isourolithin A and comparison with its urolithin A isomer: the role of cell metabolism. *Eur. J. Nutr.* 56 831–841. 26680596 10.1007/s00394-015-1131-7

[B17] Gupta R. S. Chen W. J. Adeolu M. Chai Y. (2013). Molecular signatures for the class Coriobacteriia and its different clades; proposal for division of the class Coriobacteriia into the emended order Coriobacteriales, containing the emended family Coriobacteriaceae and Atopobiaceae fam. nov., and Eggerthe. *Int. J. Syst. Evol. Microbiol.* 63 3379–3397. 10.1099/ijs.0.048371-0 23524353

[B18] Jukes T. H. Cantor C. R. (1969). *Evolution of Protein Molecules.* New York, NY: Academic Press, 21–132.

[B19] Kumar S. Stecher G. Tamura K. (2016). MEGA7: molecular evolutionary genetics analysis version 7.0 for bigger datasets. *Mol. Biol. Evol.* 33 1870–1874. 10.1093/molbev/msw054 27004904 PMC8210823

[B20] Larrosa M. García-Conesa M. T. Espín J. C. Tomás-Barberán F. A. (2010). Ellagitannins, ellagic acid and vascular health. *Mol. Aspects Med.* 31 513–539. 10.1016/j.mam.2010.09.005 20837052

[B21] Lucena T. Pascual J. Garay E. Arahal D. R. Macián M. C. Pujalte M. J. (2010). *Haliea mediterranea* sp. nov., a marine gammaproteobacterium. *Int. J. Syst. Evol. Microbiol.* 60 1844–1848. 10.1099/ijs.0.017061-0 19767360

[B22] Matthies A. Clavel T. Gutschow M. Engst W. Haller D. Blaut M. (2008). Conversion of daidzein and genistein by an anaerobic bacterium newly isolated from the mouse intestine. *Appl. Environ. Microbiol.* 74 4847–4852. 10.1128/AEM.00555-08 18539813 PMC2519357

[B23] Nuñez-Sánchez M. A. García-Villalba R. Monedero-Saiz T. García-Talavera N. V. Gómez-Sánchez M. B. Sánchez-Álvarez C. (2014). Targeted metabolic profiling of pomegranate polyphenols and urolithins in plasma, urine and colon tissues from colorectal cancer patients. *Mol. Nutr. Food Res.* 58 1199–1211. 10.1002/mnfr.201300931 24532260

[B24] Romo-Vaquero M. García-Villalba R. González-Sarrías A. Beltrán D. Tomás-Barberán F. A. Espín J. C. (2015). Interindividual variability in the human metabolism of ellagic acid: contribution of *Gordonibacter* to urolithin production. *J. Funct. Foods* 17 785–791. 10.1016/j.jff.2015.06.040

[B25] Selma M. V. González-Sarrías A. Salas-Salvadó J. Andrés-Lacueva C. Alasalvar C. Orem A. (2016). The gut microbiota metabolism of pomegranate or walnut ellagitannins yields two urolithin-metabotypes that correlate with cardiometabolic risk biomarkers: comparison between normoweight, overweight-obesity and metabolic syndrome. *Clin. Nutr.* 17 30103–30106. 10.1016/j.clnu.2017.03.012 28347564

[B26] Selma M. V. Romo-Vaquero M. García-Villalba R. González-Sarrías A. Tomás-Barberán F. A. Espín J. C. (2015). The human gut microbial ecology associated with overweight and obesity determines ellagic acid metabolism. *Food Funct.* 7 1769–1774. 10.1039/c5fo01100k 26597167

[B27] Selma M. V. Tomas-Barberan F. A. Beltran D. García-Villalba R. Espin J. C. (2014a). *Gordonibacter urolithinfaciens* sp. nov., a urolithin-producing bacterium isolated from the human gut. *Int. J. Syst. Evol. Microbiol.* 64 2346–2352. 10.1099/ijs.0.055095-0 24744017

[B28] Selma M. V. Beltrán D. García-Villalba R. Espín J. C. Tomás-Barberán F. A. (2014b). Description of urolithin production capacity from ellagic acid of two human intestinal *Gordonibacter* species. *Food Funct.* 5 1779–1784. 10.1039/c4fo00092g 24909569

[B29] Stackebrandt E. Goebel B. M. (1994). Taxonomic note: a place for DNA-DNA reassociation and 16S rRNA sequence analysis in the present species definition in bacteriology. *Int. J. Syst. Bacteriol.* 44 846–849.

[B30] Thawornkuno C. Tanaka M. Sone T. Asano K. (2009). Biotransformation of daidzein to equol by crude enzyme from *Asaccharobacter celatus* AHU1763 required an anaerobic environment. *Biosci. Biotechnol. Biochem.* 73 1435–1438. 10.1271/bbb.80908 19502755

[B31] Tomás-Barberán F. A. García-Villalba R. González-Sarrías A. Selma M. V. Espín J. C. (2014). Ellagic acid metabolism by human gut microbiota: consistent observation of three urolithin phenotypes in intervention trials, independent of food source, age, and health status. *J. Agric. Food Chem.* 62 6535–6538. 10.1021/jf5024615 24976365

[B32] Tomás-Barberán F. A. González-Sarrías A. García-Villalba R. Núñez-Sánchez M. A. Selma M. V. García-Conesa M. T. (2016a). Urolithins, the rescue of “old” metabolites to understand a “new” concept: metabotypes as a nexus between phenolic metabolism, microbiota dysbiosis and host health status. *Mol. Nutr. Food Res.* 61 1–74. 10.1002/mnfr.201500901 27158799

[B33] Tomás-Barberán F. A. Selma M. V. Espín J. C. (2016b). Interactions of gut microbiota with dietary polyphenols and consequences to human health. *Curr. Opin. Clin. Nutr. Metab. Care* 19 471–476. 10.1097/MCO.0000000000000314 27490306

